# Osteoarthritis prevalence and modifiable factors: a population study

**DOI:** 10.1186/s12889-015-2529-0

**Published:** 2015-11-30

**Authors:** Ronald Plotnikoff, Nandini Karunamuni, Ellina Lytvyak, Christopher Penfold, Donald Schopflocher, Ikuyo Imayama, Steven T. Johnson, Kim Raine

**Affiliations:** Priority Research Centre for Physical Activity and Nutrition, University of Newcastle, Callaghan, NSW 2308 Australia; School of Public Health, University of Alberta, Edmonton, AB Canada; Fred Hutchinson Cancer Research Center Seattle, Washington, USA; Centre for Nursing and Health Studies, Athabasca University, Athabasca, AB Canada

**Keywords:** Knee osteoarthritis, Hip osteoarthritis, Prevalence, Risk-factors

## Abstract

**Background:**

This study’s objectives were to investigate the prevalence of self-reported knee and hip osteoarthritis (OA) stratified by age and sex and to examine the association of modifiable factors with knee and hip OA prevalence. The study was conducted using randomly sampled data gathered from four communities in the province of Alberta, Canada.

**Methods:**

A large adult population sample (*N* = 4733) of individuals ≥18 years were selected. Health-related information was collected through telephone interviews and community measurement clinics for which a sub-sample (*N* = 1808) attended. Participants self-reported OA during telephone interviews. Clinic interviews further assessed if the diagnosis was made by a health care professional. Statistical analyses compared prevalence of OA between sexes and across age categories. Associations between modifiable factors for OA and the prevalence of knee and hip OA were assessed using binary logistic regression modelling.

**Results:**

Overall prevalence of self-reported OA in the total sample was 14.8 %, where 10.5 % of individuals reported having knee OA and 8.5 % reported having hip OA. Differences in prevalence were found for males and females across age categories for both knee and hip OA. In terms of modifiable factors, being obese (BMI >30 kg/m2) was significantly associated with the prevalence of knee (OR: 4.37; 95 % CI: 2.08,9.20) and hip (OR: 2.52; 95 % CI: 1.17,5.43) OA. Individuals who stand or walk a lot, but do not carry or lift things during their occupational activities were 2.0 times less likely to have hip OA (OR: 0.50; 95 % CI: 0.26,0.96). Individuals who usually lift or carry light loads or have to climb stairs or hills were 2.2 times less likely to have hip OA (OR: 0.45; 95 % CI: 0.21,0.95). The odds of having hip OA were 1.9 times lower in individuals consuming recommended or higher vitamin C intake (OR: 0.52; 95 % CI: 0.29,0.96). Significant differences in prevalence were found for both males and females across age categories.

**Conclusion:**

The prevalence of knee and hip OA obtained in this study is comparable to other studies. Females have greater knee OA prevalence and a greater proportion of women have mobility limitations as well as hip and knee pain; it is important to target this sub-group.

## Background

Osteoarthritis (OA) is the most common articular disease of the developed world and a leading cause of chronic disability, mostly as a consequence of knee OA and/or hip OA [1–3]. The economic costs of OA are high, including those related to treatment, for individuals and their families who must adapt their lives to the disease, and those due to lost work productivity [4, 5]. The prevalence of hand, knee, or hip joint OA has increased from 21 million in 1995 to an estimated 27 million among United States (US) adults [3]. Such increases are likely due to aging of the population and the rising prevalence of obesity [2].

OA has a multi-factorial etiology, with different sets of factors associated with its incidence [1, 5]. Factors associated with OA have been broadly divided into person-level factors and joint-level factors [2]. Person-level factors include age, sex, obesity, genetics, race/ethnicity and diet. Joint-level factors refer to factors that are unique to a particular joint such as, injury, activity, type of occupation, and muscle strength [2]. Factors associated with OA have also been classified as those that relate to OA development and those relating to disease progression. In terms of knee OA, Doherty [6], reports factors such as age, sex, occupation, weight status and recreational activity can play a role in the development of OA, and weight status and dietary factors may play a role in its progression.

When considering non-modifiable factors for OA, age and sex are the strongest predictors. For example, women are at greater risk for developing knee and hip OA compared to their male counterparts [5, 7, 8]. Hormonal factors, reduced volume of cartilage in the knee, and the fact that women are more likely to self-report have been considered as explanatory factors [5, 7, 8].

Age is a significant contributor to the sex differences in prevalence of OA, where females tend to have more severe knee and hand OA than men, particularly after menopausal age [1, 8]. Age is one of the strongest non-modifiable factors for OA [1, 5], where this relationship is likely related to a combination of changes in the capacity for joint tissues to adapt to biomechanical stresses [2].

Obesity is a strong modifiable risk factor for the development of knee OA [1, 9], but less so for hip OA [2]. In a meta-analysis, those who were obese or overweight were nearly three times as likely to report knee OA [9]. The effects of obesity on OA are through both mechanical and systemic mechanisms. Obesity can exert an increased load as a consequence of increased body weight, however there may be differential systemic effects depending on the degree of fat versus lean mass [2, 10], involving the activity of adipocytokines [11].

Other modifiable factors of OA include occupation, dietary factors and physical activity [2, 5]. For example, repetitive joint loading through kneeling or squatting have been shown to be associated with an increased risk of knee OA [2, 12], and this risk is even greater for those who are overweight [2]. Furthermore, occupational lifting and prolonged standing have also been most strongly associated with hip OA [2, 12].

A number of studies have examined the role of vitamins (such as vitamins D and C) in OA [2, 13–16]. Mechanistically, it is thought that vitamin C may serve to decrease cartilage loss in the joints while low vitamin D intake and reduced circulating serum vitamin D may confer an increased risk of knee OA [13].

The benefits of physical activity for OA are well-established [17], with national service organizations promoting active lifestyles, including walking for individuals with OA [18]. However, most individuals with knee OA do not meet recommended physical activity guidelines [19]. Findings from a recent study has reported most people with knee OA are capable of walking at the recommended intensity needed to meet physical activity guidelines, and their knee pain has little impact on their level of physical activity [20].

Factors associated with OA could also interact in complex ways. For example, healthy lifestyle behaviours may reduce the age-related onset of OA, and there can also be additional multifaceted associations between factors associated with OA. Considering the rising prevalence of OA in the population, identifying modifiable factors associated with OA is important to guide the development of effective interventions. Currently, there appears to be a paucity of data, particularly for Canada [21].

Using a large population sample consisting of random community samples, the objectives of this study were to: (i) investigate the prevalence of self-reported knee and hip OA stratified by age and sex; and, (ii) to examine the association of modifiable factors of body mass index (BMI), occupational factors, physical activity, vitamin C and D -intake, with self-reported knee OA and hip OA.

## Methods

**Fig. 1:**
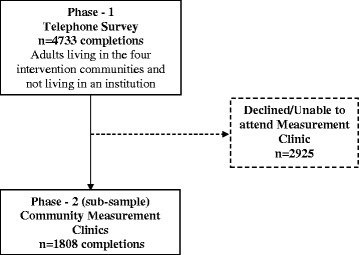
Flow of participants from Phase 1 to Phase 2

Data were gathered as part of the 2009–10 assessment period of the Healthy Alberta Communities (HAC) project, the details of this project and information on its sampling frame are reported elsewhere [22, 23]. Briefly, adults of four communities in the province of Alberta, Canada were randomly sampled (see Fig. 1). Data collection from the study participants took place in distinct phases. Phase 1 consisted of Computer Assisted Telephone Interviewing protocol, where survey data were gathered from 4733 individuals. From these individuals, a subsample of 1808 (38.2 %) agreed to take part in community measurement clinics that collected clinical measures and additional self-reported health-related information (Phase 2). Inclusion criteria for the HAC project (Phase 1) were men and women of the intervention communities who were aged 18 years and older and not living in an institution. Out of these participants, pregnant women and persons in wheelchairs were excluded from Phase 2 of the study. Data from Phase 1 (telephone-based assessment) and Phase 2 (clinical-based assessments) are presented in the analysis of this paper.

### Ethics

The study was approved by Research Ethics Boards at the University of Alberta. All data were collected directly from participants. Potential respondents to the telephone survey were informed of the purpose of the survey and asked to participate. Participation was completely voluntary. Agreement to participate served as verbal informed consent for Phase 1. For Phase 2 (i.e., subsample of those completing Phase 1), participants were asked to sign informed consent for the collection of physical measures and blood.

### Measures

In Phase 1 of the study, during the phone interviews, participants were asked to report their age and sex, as well as to respond ‘yes’ or ‘no’ to the questions, “*Do you have knee osteoarthritis?”* and “*Do you have hip osteoarthritis?”* The options “*Do not know*” or “*Refuse to answer*” were also provided. This is a standard self-report measure employed in the Canadian Community Health Survey (CCHS) [24]. Categories of BMI were generated using participant self-reported height and weight estimates (objectively measured BMI was also obtained during the clinical assessments using a standardized protocol and equipment [23]). Physical activity level was assessed with a brief validated measure where participants were also asked to describe their main daily activities as*: sit and don’t walk; stand or walk quite a lot but do not carry or lift things; usually lift or carry light loads or have to climb stairs or hills often; do heavy work or carry very heavy loads* [24]*.* The validated Godin-leisure time physical activity instrument, was employed to assess leisure-time physical activity [25]. Weekly frequencies of strenuous, moderate, and mild activities were multiplied by nine, five, and three, respectively, and then summed to obtain total weekly leisure activity scores [25]. Using validated cut-points, individuals having a weekly leisure activity score ≥24 were classified as active for substantial health benefit and individuals with scores ≤23 were classified as inactive [26].

In Phase 2 of the study (clinic interviews) participants (*n* = 1808) answered ‘yes’ or ‘no’ to a five-item questionnaire relating to knee OA and mobility limitations, using a validated questionnaire [27]. Participants were asked: *During the past 4 weeks, have you had knee pain on most days?; During the past 4 weeks, have you had knee pain while climbing down stairs or walking down slopes?; During the past 4 weeks, have you had swelling in one or both knees?; Do you have knee osteoarthritis?; If you do, was the diagnosis made by a rheumatologist or a general practitioner* [27]*?* Further, participants responded ‘yes’ or ‘no’ to the following questions relating to hip OA and mobility limitations: *During the past 4 weeks, have you had hip pain (groin or upper thigh) on most days?; During the past 4 weeks, have you had hip pain while climbing down stairs or walking down slopes?; During the past 4 weeks, have you noticed any limitation in the range of motion of one or both hips?; Do you have hip osteoarthritis? If you do, was the diagnosis made by a rheumatologist or a general practitioner* [27]*.* Participants who responded ‘yes’ to (i) having OA through the phone survey, (ii) having OA through the clinic survey, and (iii) indicated their OA was diagnosed by a general practice physician or rheumatologist was coded as the study’s robust indicator of OA.

During the Phase 2 assessments, Vitamin C intake was estimated from the Block – Brief 2000 Food Frequency Questionnaire [28], and participants were categorised as meeting recommendations versus not meeting recommendations according to the daily intake of <90 mg/day for men; and <75 mg/day for women [29]. Vitamin D intake was also estimated from the Block – Brief 2000 Food Frequency Questionnaire and categorised as meeting versus not meeting the recommended daily intake of 600 IU/day for 19–70 years, and <800 IU/day for 70+ years [30].

### Data analyses

Descriptive statistics were calculated for all study variables. Age was stratified according to classification used in the National Health Interview Survey [31], using the categories: 18–44; 45–64 and ≥65 years. BMI was calculated as weight in kilograms divided by height in meters squared, and BMI categories were defined as: underweight/normal (BMI <25); overweight (25 ≤BMI <30); obese (30 ≤BMI) [32].

Descriptive analysis was performed to identify the overall OA prevalence in the study sample, and the prevalence was stratified by age and sex for both knee and hip OA separately. Prevalence was also compared between sexes and across age categories.

Estimates of BMI from self-reported height and weight and the objectively measured height and weight were compared using Bland and Altman limits of agreement [33], as well as bivariate Pearson correlations. Among the sub-group of participants (*n* = 1808) who provided data from both phone interviews and community clinics, the reliability of self-reported OA through the single-item phone interview was compared with the five-item clinic interview measure using Cohen’s Kappa coefficient [34]. As suggested by Landis and Koch [35], a kappa of 0.40–0.75 was considered to represent intermediate to good agreement.

Associations between modifiable factors associated with OA and the prevalence of knee and hip OA were assessed using binary logistic regression modelling [study objective (ii)]. The dependent outcome variable was prevalence of robust knee OA or hip OA constructed as a ‘yes/no’ dichotomous indicator. Based on existing literature identifying potential factors associated with OA, independent explanatory variables for the multivariate models included: age, sex, weight status (BMI), occupational activity, leisure-time physical activity, vitamin C-intake and vitamin D-intake. Effects by age and sex were estimated using stratified logistic regression models. All the covariates included in our analyses had less than 5 % missing data, with the exception of the leisure time physical activity measure which had 7.1 % missing cases. Taking into account sufficiently large sample size, complete case analysis was applied [36–38].

Survey weights were calculated using year specific community age and sex counts supplied by the Alberta government for post-stratification. In addition, 300 bootstrap weights were generated to account for difference in sample characteristics (i.e., community of residence, age category, smoking, BMI, physical activity level, fruit and vegetable consumption, and self-reported health) between the Phase 1 and Phase 2 group. All analyses were performed using sample and bootstrap weights.

## Results

Demographic characteristics of the sample are displayed in Table 1. The average age of the participants was 52.5 (±16.5) years and 55.2 % of them had a university degree. Of the participants, 32.6 % were male. The overall prevalence of self-reported OA (knee or hip) in the total sample (*N* = 4733) was 14.8 %. Out of these individuals, 10.5 % self-reported having knee OA and 8.5 % self-reported having hip OA with approximately 3 % self-reported having both knee and hip OA. For knee OA, 2.1 % gave the answer *“Do not know”* and 0.03 % refused to answer. For hip OA, these values were 1.7 and 0.03 % respectively.

**Table 1:** Demographic characteristics of the overall study sample (*N* = 4733)

	N(%) or mean ± SD
*Sex*	
Male	1542 (32.6)
Female	3189 (67.4)
*Age, years*	52.5 ± 16.5
*Education*	
- No university	2095 (44.5)
- University	2611 (55.2)
*Sports participation* ^a^	
- Weekly leisure activity score ≥24	651 (38.3)
- Weekly leisure activity score ≤23	1049 (61.7)
*Daily activity*	
- Sit/not walk	1099 (23.6)
- Stand/walk	1988 (42.7)
- Light work load	1304 (28.0)
- Heavy work loads	270 (5.8)
Level of combined household income (before tax), above cut-off point^b^	3700 (86.5)
Knee osteoarthritis self-reported	482 (10.5)
Hip osteoarthritis self-reported	395 (8.5)
Either knee or hip osteoarthritis self-reported	679 (14.8)
BMI self-reported, (kg/m^2^)	26.81 ± 5.51
BMI measured, (kg/m^2^)^a^	28.28 ± 5.98
Overweight measured^a^	630 (34.8)
Obese measured^a^	591 (32.7)

Data are based on non-weighted estimates

^a^These measured variables were obtained during clinic interviews that were attended by 1808 participants

^b^The cut-off point for low-income is Low Income Cut-Offs (LICO) were calculated for each participant based on family size and belonging to the particular community [53]

Prevalence (weighted estimates) of both knee and hip OA stratified by age and sex are displayed in Table 2. For knee OA, the prevalence was 6.3 % for males and 8.9 % for females (t = 3.38; *p* = 0.001). For hip OA, the prevalence was 4.4 % for males and 7.6 % for females (t = 4.64; *p* = 0.001). For robust measures of knee and hip OA, obtained through clinic surveys, the prevalence of knee OA was 4.4 % in males and 6.7 % in females (t = 1.6; *p* = 0.112). For hip OA, the rates were 2.9 for males, and 4.1 for females (t = 1.0; *p* = 0.325).

**Table 2:** Prevalence of knee and hip osteoarthritis by age and sex

	Males 49.9 % (*N* = 2364)	Females 50.0 % (*N* = 2367)	Differential significance between sexes, *p*
*Knee osteoarthritis* ^a^			
All ages	6.3 (146)	8.9 (206)	0.001
18–44	1.5 (17)	1.7 (19)	0.651
45–64	8.1 (62)	10.9 (78)	0.063
>65	18.8 (61)	24.3 (97)	0.069
*Hip osteoarthritis* ^a^			
All ages	4.4 (102)	7.6 (176)	<0.001
18–44	1.3 (15)	0.7 (7)	0.119
45–64	4.7 (36)	8.5 (61)	0.003
>65	14.2 (47)	24.6 (99)	<0.001
*Knee osteoarthritis* ^b^			
All ages	4.4 (23)	6.7 (33)	0.112
18–44	3.2 (8)	1.1 (3)	0.121
45–64	3.9 (7)	9.5 (15)	0.042
>65	10.3 (8)	17.2 (14)	0.210
*Hip osteoarthritis* ^b^			
All ages	2.9 (15)	4.1 (20)	0.325
18–44	2.8 (7)	0.3 (1)	0.030
45–64	2.0 (4)	5.5 (9)	0.090
>65	6.1 (5)	13.0 (10)	0.145
*Knee OR Hip osteoarthritis* ^b^			
All ages	6.5 (34)	8.8 (43)	0.173
18–44	5.1 (12)	1.5 (3)	0.025
45–64	5.9 (11)	12.0 (19)	0.049
>65	13.4 (10)	24.4 (19)	0.085

Data are based on weighted estimates

^a^Values reported are self-reported data based on phone interviews

^b^Values reported are based on robust values. Robust values represent participants responding ‘yes’ to having OA through both the phone survey and clinic survey, and indicating their OA was diagnosed by a general practice physician or rheumatologist

The reliability (Cohen’s Kappa coefficient) of self-reported OA through the phone interview compared with the five-item measure during the clinic interview was κ = 0.73 for knee OA, and κ = 0.68 for hip OA. Based on Phase 2 data, the correlations between the prevalence of: knee osteoarthritis and ‘knee pain on most days’ was 0.43 (*p* <.01); knee osteoarthritis and knee swelling was 0.38 (*p* <.01); and hip osteoarthritis and ‘hip pain on most days’ was 0.45; (*p* <.01). The average BMI obtained from self-reported height and weight compared to measured BMI was 26.8 ± 5.5 kg/m^2^ and 28.3 ± 6.0 kg/m^2^ respectively. The mean difference between self-reported and measured BMIs was −1.25 kg/m^2^ (limits of agreement: −5.28 and 2.78). The Pearson correlations between these two measures was r = 0.93.

Significant differences in prevalence were found for both males and females across age categories (see Table 3). Prevalence was significantly related to older age for both males and females for knee OA (females: *χ*^2^ = 188.9, *p* <0.001; males: *χ*^2^ (2) =136.5; *p* <0.001) and for hip OA (females *χ*^2^ = 238.8, *p* <0.001; males *χ*^2^ = 104.2, *p* <0.001).

**Table 3:** Prevalence^a^ of OA and mobility limitations stratified by age and sex

	Males				Females			
	18-44 years	45-64 years	>65 years	*χ* ^2^- value; *p*-value	18-44 years	45-64 years	>65 years	*χ* ^2^- value; *p*-value
Osteoarthritis - phone survey (*n* = 4733)								
*Knee OA (yes)*	0.8	2.8	2.7	136.452; *p* <0.001	0.9	3.6	4.4	188.940; *p* <0.001
*Hip OA (yes)*	0.7	1.6	2.1	104.127; *p* <0.001	0.3	2.8	4.5	238.793; *p* <0.001
Osteoarthritis – clinic survey (*n* = 1808)								
*Knee pain (yes)*	8.2	7.7	3.1	4.609; *p* = 0.1	5.4	9.0	6.1	38.217; *p* <0.001
*Knee pain climbing stairs/walking down slope (yes)*	7.7	11.9	4.4	38.793; *p* <0.001	8.5	12.5	8.0	42.096; *p* <0.001
*Knee OA? (yes)*	1.3	2.6	2.5	35.710: *p* <0.001	0.6	4.8	4.8	80.594; *p* <0.001
*Knee/s swelling (yes)*	5.7	3.2	1.9	1.823; *p* = 0.402	3.3	5.1	2.8	13.406; *p* <0.001
*Knee OA diagnosed? (yes)*	2.3	4.8	3.6	31.149; *p* <0.001	0.8	7.5	7.7	78.988; *p* <0.001
*Hip pain (yes)*	2.6	5.9	4.0	54.460; *p* <0.001	4.1	9.3	7.4	71.951; *p* <0.001
*Hip pain climbing stairs/walking down slope (yes)*	2.2	6.1	3.7	57.617; *p* <0.001	3.3	8.3	6.2	63.296; *p* <0.001
*Hip range of motion limited (yes)*	1.5	4.4	2.2	32.670; *p* <0.001	2.7	8.0	4.9	55.798; *p* <0.001
*Hip OA? (yes)*	1.0	1.2	1.7	25.371; *p* <0.001	0.5	3.3	5.3	97.776; *p* <0.001
*Hip OA diagnosed? (yes)*	2.1	2.7	2.7	19.970; *p* <0.001	0.6	5.2	9.1	98.757; *p* <0.001

^a^Prevalence values are percentages

The results of assessments relating to OA and mobility limitations conducted during clinic interviews are presented in Table 3. Among females, for the age categories 18–44 years, 45–64 years and >65 years, 5.4, 9.0 and 6.1 % reported having knee pain. The corresponding values for the three age groups having hip pain were 4.1, 9.3 and 7.4 %. The values for males for the same age categories were 8.2, 7.7 and 3.1 % for knee pain and 2.6, 5.9 and 4.0 % for hip pain (Table 3).

Results of binary logistic regression modelling for both knee and hip OA are displayed in Table 4 [study objective (ii)]. Among the overall sample, being obese (BMI >30 kg/m^2^) was significantly associated with the prevalence of knee (OR: 4.37; 95 % CI: 2.08, 9.20; *p* <0.001) and hip (OR: 2.52; 95 % CI: 1.17, 5.43; *p* = 0.018) OA.

**Table 4:** Binary logistic regression modelling of osteoarthritis

Total sample^c^	Odds ratio (95 % CI); p-value	
	*Knee osteoarthritis*	*Hip osteoarthritis*
*BMI – clinic assessed*		
Under/ normal weight (*n* = 508)	–	–
Overweight (*n* = 547)	1.94 (95 % CI: 0.87, 4.32); *p* = 0.105	1.45 (95 % CI: 0.64, 3.27); *p* = 0.369
Obese (*n* = 509)	4.37 (95 % CI: 2.08, 9.20); p <0.001	2.52 (95 % CI: 1.17, 5.43); p = 0.018
*Daily activity*		
Sit/not walk (*n* = 359)	–	–
Stand/walk (*n* = 700)	1.11 (95 % CI: 0.62, 2.00); *p* = 0.725	0.50 (95 % CI: 0.26, 0.96); p = 0.038
Light work load (*n* = 433)	0.72 (95 % CI: 0.36, 1.44); *p* = 0.354	0.45 (95 % CI: 0.21, 0.95); p = 0.037
Heavy workloads (*n* = 72)	1.00 (95 % CI: 0.27, 3.69); *p* = 0.995	1.51 (95 % CI: 0.47, 4.79); *p* = 0.488
*Sports*		
Weekly leisure activity (score ≤23, %) (*n* = 682)	–	–
Weekly leisure activity (score ≥24, %) (*n* = 882)	0.67 (95 % CI: 0.41, 1.11); *p* = 0.124	1.21 (95 % CI: 0.69, 2.12); *p* = 0.508
*Vitamin C - clinic interview* ^a^		
Less (*n* = 928)	–	–
Recommendation (*n* = 636)	1.35 (95 % CI: 0.82, 2.21); *p* = 0.237	0.52 (95 % CI 0.29, 0.96); p = 0.035
*Vitamin D - clinic interview* ^b^		
Less (*n* = 1401)	–	–
Recommendation (*n* = 163)	1.48 (95 % CI: 0.67, 3.28); *p* = 0.328	1.73 (95 % CI 0.67, 4.44); *p* = 0.258
Sex: Male^d^		
*BMI – clinic assessed*		
Under/ normal weight (*n* = 136)	–	–
Overweight (*n* = 211)	1.90 (95 % CI: 0.60, 6.08); *p* = 0.277	0.81 (95 % CI: 0.28, 2.32); *p* = 0.690
Obese (*n* = 179)	2.90 (95 % CI: 0.93, 9.02); *p* = 0.066	0.99 (95 % CI: 0.35, 2.82); *p* = 0.992
*Daily activity*		
Sit/not walk (*n* = 142)	–	–
Stand/walk (*n* = 214)	1.42 (95 % CI: 0.58, 3.46); *p* = 0.441	0.59 (95 % CI: 0.22, 1.65); *p* = 0.317
Light work load (*n* = 131)	1.02 (95 % CI: 0.37, 2.81); *p* = 0.968	0.45 (95 % CI: 0.14, 1.49); *p* = 0.193
Heavy workloads (*n* = 39)	0.47 (95 % CI: 0.05, 4.41); *p* = 0.508	1.32 (95 % CI: 0.34, 5.17); *p* = 0.691
*Sports*		
Weekly leisure activity (score ≤23, %) (*n* = 209)	–	–
Weekly leisure activity (score ≥24, %) (*n* = 317)	0.59 (95 % CI: 0.28, 1.23); *p* = 0.156	1.43 (95 % CI: 0.58, 3.52); *p* = 0.439
*Vitamin C - clinic interview* ^a^		
Less (*n* = 337)	–	–
Recommendation (*n* = 189)	0.99 (95 % CI: 0.46, 2.15); *p* = 0.981	0.48 (95 % CI 0.18, 1.31); *p* = 0.152
*Vitamin D - clinic interview* ^b^		
Less (*n* = 489)	–	–
Recommendation (*n* = 37)	0.52 (95 % CI: 0.07, 3.73); *p* = 0.512	0.00; *p* = 0.998
Sex: Female^d^		
*BMI – clinic assessed*		
Under/ normal weight (*n* = 372)	–	–
Overweight (*n* = 336)	1.83 (95 % CI: 0.59, 5.71); *p* = 0.295	2.79 (95 % CI: 0.72, 10.78); *p* = 0.137
Obese (*n* = 330)	6.72 (95 % CI: 2.45, 18.45); p <0.001	6.67 (95 % CI: 1.84, 24.21); p = 0.004
*Daily activity*		
Sit/not walk (*n* = 217)	–	–
Stand/walk (*n* = 486)	0.96 (95 % CI: 0.43, 2.15); *p* = 0.928	0.53 (95 % CI: 0.21, 1.30); *p* = 0.165
Light work load (*n* = 302)	0.55 (95 % CI: 0.21, 1.43); *p* = 0.219	0.46 (95 % CI: 0.16, 1.26); *p* = 0.131
Heavy workloads (*n* = 33)	2.46 (95 % CI: 0.45, 13.45); *p* = 0.298	1.74 (95 % CI: 0.18, 17.29); *p* = 0.635
*Sports*		
Weekly leisure activity (score ≤ 23, %) (*n* = 473)	–	–
Weekly leisure activity (score ≥ 24, %) (*n* = 565)	0.86 (95 % CI: 0.42, 1.72); *p* = 0.662	1.08 (95 % CI: 0.50, 2.33); *p* = 0.848
*Vitamin C - clinic interview* ^a^		
Less (*n* = 591)	–	–
Recommendation (*n* = 447)	1.73 (95 % CI: 0.88, 3.42); 0.113	0.61 (95 % CI 0.27, 1.36); *p* = 0.226
*Vitamin D - clinic interview* ^b^		
Less (*n* = 912)	–	–
Recommendation (*n* = 126)	2.07 (95 % CI: 0.82, 5.22); *p* = 0.121	3.35 (95 % CI: 1.14, 9.88); p = 0.028

The first-listed category for each variable is the reference category

Dependent variable was robust self-reported knee OA or hip OA. Robust values represent individual’s self-reported OA (during phone interview and during clinic interview), as well as being diagnosed as having OA during clinic interviews

^a^Vitamin C recommendations were based on the Institute of Medicine (2006): adult males 90 mg/day; adult females 75 mg/day

^b^Vitamin D recommendations were based on the Institute of Medicine (2011): adults <70 years 600 IU/day and adults ≥70 years 800 IU/day

Analyses adjusted for age and sex

^c^Analyses were controlled for age and sex

^d^Analyses were controlled for age

Among the overall sample, individuals who stand or walk quite a lot, but do not carry or lift things during their occupational activities were 2.0 times less likely to have hip OA (OR: 0.50; 95 % CI: 0.26, 0.96; *p* = 0.038). Further, persons who usually lift or carry light loads or have to climb stairs or hills were 2.2 times less likely to have hip OA (OR: 0.45; 95 % CI: 0.21, 0.95; *p* = 0.037). Additionally, the odds of having hip OA were 1.9 times lower in individuals consuming recommended or higher vitamin C intake (OR: 0.52; 95 % CI 0.29, 0.96; *p* = 0.035).

Stratified models indicated that among females, being obese was significantly related with knee OA prevalence (OR: 6.72; 95 % CI: 2.45, 18.45; *p* <0.001) and hip OA prevalence (OR: 6.67; 95 % CI: 1.84, 24.21; *p* = 0.004). The odds of hip OA were 3.35 times higher among females with vitamin D intakes at recommended levels or higher (OR: 3.35; 95 % CI: 1.14, 9.88; *p* = 0.028). None of the variables were significant for the males, except for age. Level of physical activity was not significantly associated with knee or hip OA prevalence for either sex.

## Discussion

The objectives of the current study were to investigate the prevalence of knee and hip OA and to examine the association of modifiable factors with knee and hip OA prevalence among a large population-based sample. Here we report the 14.8 % prevalence of knee or hip OA by self-report is comparable to other studies. In the US, the overall OA prevalence is 13.9 % for adults aged 25 and older [3]. A review of several investigations in Canada has reported the overall prevalence ranges between 7.5 and 14.7 % [21].

In agreement with the current literature [1, 2], our study found that age is associated with the prevalence of both knee and hip OA for both males and females. The prevalence of OA was also higher among females, which is comparable to findings from other studies [1, 5, 7, 8]. Further, a greater proportion of women had higher percentages on all six measures relating to mobility limitations, hip and knee pain, as well as swelling [3].

In terms of knee OA, females had greater prevalence compared to males which is consistent with the current literature. For example, in a review by Lawrence, and colleagues [3], knee OA prevalence among US women ranged from 4.9 to 18.7 %. In this review, some of the studies included older age groups (such as ≥60 years), whereas our sample consisted of individuals 18+, which may account for the lower proportions (i.e., younger individuals are less likely to be affected by OA) [3]. Differences in survey methods used, ethnicity and other sampling issues may also affect prevalence values across various surveys [1, 39].

Considering hip OA, our reported hip OA prevalences of 4.4 and 7.6 % for men and women respectively are also comparable to the current literature. For instance, the Johnston County OA Project [40], that surveyed individuals ≥45 years of age reported the rates for hip OA among men and women to be 8.7 and 9.3 % respectively. A more recent study of individuals ≥60 years of age living in Spain [41], documented a prevalence of 6.7 % in men and 8.0 % in women.

In our study, the robust measure (i.e., percentage of participants who responded ‘yes’ to having OA through both the phone survey and clinic survey, and indicated their OA was diagnosed by a general practice physician or rheumatologist), yielded lower prevalence for both knee and hip OA (than the single-item, self-report measures). The diagnosis of symptomatic radiographic OA by a physician or rheumatologist takes into consideration both structural change and joint pain or discomfort [1], whereas self-report measures may only involve subjective assessments of joint pain. Therefore, a lower prevalence when using the robust measure is somewhat expected. Further, when considering the study’s robust measure, sex differences in prevalence was not significant for either knee or hip OA prevalence. According to the literature, self-report methods for detecting OA are known to exaggerate sex differences in prevalence due to reporting bias; women may be more likely to self-report OA [8].

In our logistic regression analyses, for the overall sample and for females, being obese was strongly associated with knee and hip OA prevalence. Obesity is a well-established modifiable risk factor of knee OA [1, 9, 42]. The lifetime risk of developing symptomatic knee OA is approximately 40 % in men and 47 % in women, with higher risks among those who are obese [43], and studies have shown that decreasing BMI by two units or more over 10 years is associated with a 50 % lower risk of developing symptomatic knee OA among women [44]. Further, duration of exposure to high BMI during adulthood confers risk of incident knee OA, suggesting the importance of weight control throughout life as a means of primary prevention of knee OA [45]. Although our study showed that obesity is also associated with hip OA, according to the literature, the relationship between obesity and hip OA is weaker than with knee OA [1, 2].

Certain occupations have been shown to be associated with an increased risk of OA. Most studies report significantly greater risk of OA in individuals whose occupations involve activities with high physical demands [1, 2, 12, 46]. Among occupational activity, our study found that individuals who spend time standing or walking, as well as lifting or carrying light loads and climb stairs or hills had a significantly lower risk of hip OA. While our study found no association for physically demanding work, the association of standing, walking and carrying light loads with a significantly lower risk of hip OA may indicate benefits of physical activity; considering that benefits of being active is well-established for OA [17]. However, we caution this interpretation considering our results cannot be used to infer cause and effect.

In our logistic regression model, physical activity was not associated with OA prevalence, which may be explained by the cross-sectional design. However, 61.7 % of the participants reported being inactive (weekly leisure activity score ≤23), whilst those with knee and hip OA were 79.0 and 75.7 % inactive, respectively (compared to 58.5 % of persons without OA being inactive). The percentage of inactive individuals we observed is higher than a US survey that found 44 % of persons with arthritis were inactive (based on self-report measures) compared to 36 % of adults without arthritis [47]. Our study’s inactivity prevalence is concerning, considering physical activity can reduce pain, improve physical performance, as well as provide multiple other benefits of physical activity [17, 18].

In terms of dietary factors, the overall sample’s odds of hip OA were 1.9 times lower in individuals consuming vitamin C intakes at or above recommended levels (<90 mg/day for men, <75 mg/day for women) [48]. While there have been conflicting results in terms of the effect of vitamin C on knee OA [2, 5, 14, 15], only a limited number of studies have specifically examined the relationship between hip OA and vitamin C intake. Engstrom, and colleagues [49], conducted a prospective population-based study that explored the relationship between intake of antioxidants and incidence of severe hip and/or knee OA, and reported that high dietary intake of vitamin C was significantly associated with the incidence of hip OA. However, in a 2-week crossover randomized trial, Jensen [50], found that pain was reduced during vitamin C treatment of individuals with radiographically verified OA of the knee and/or hip. Other studies have indicated no convincing evidence that vitamin C is effective in the treatment of any type of arthritis [51]. Further studies are needed to understand these conflicting findings.

In our study, the odds of hip OA were 3.4 times higher in females consuming recommended or higher vitamin D intakes (19–70 years <600 IU /day, 70+ years <800 IU/day) [48]. Studies with specific focus on the association between vitamin D intake and hip OA are also very limited. One study that examined the relationship between serum vitamin D concentrations and incident radiographic hip OA among elderly women demonstrated that low serum levels of vitamin D may be associated with incident changes of radiographic hip OA characterized by joint space narrowing [52]. Our finding that only females are negatively affected by vitamin D is interesting and warrants further investigation.

### Strengths and limitations

The strengths of this study include the use of a large random sample across four Canadian communities, and the use of validated measures. A further strength is the generalizability of the sample which was randomly selected in Phase 1. Further, population-based weights were included in the analyses and bootstrapping techniques were applied to enhance the population representativeness. Limitations of the study include its cross-sectional design and the use of some self-report measures. However, Phase 2 five-item knee and hip OA measures had intermediate/good agreement with respective single-item measures from Phase 1 [35], and the self-reported and measured BMIs were highly correlated 0.93. Another limitation of our study is that we were unable to collect information regarding the specific source (oral, written, x-ray) of verification provided by their physician/rheumatologist. Additionally, assessing further factors associated with OA would have strengthened our study. For example, joint injury and resultant joint tissue destruction and loss could contribute to the development of OA [1]. Further, as vitamin D can also be obtained from sun exposure, it would have been beneficial to quantify the duration of participants’ exposure to sunlight. Including these and other factors may have given more insight into the roles played by these modifiable factors. Despite the reported limitations, this study used a large representative population sample to estimate the prevalence of OA and examine in multivariate models a host of potential modifiable factors of OA. These results will help guide practice and future research**.**

## Conclusions

The prevalence of knee and hip OA obtained in this study is comparable to other studies. Females have greater knee OA prevalence and a greater proportion of women have mobility limitations as well as hip and knee pain; it is important to target this sub-group.
